# Persistent hepatic IFN system activation in HBV-HDV infection determines viral replication dynamics and therapeutic response

**DOI:** 10.1172/jci.insight.162404

**Published:** 2023-05-08

**Authors:** Takeshi Chida, Yuji Ishida, Sho Morioka, Go Sugahara, Christine Han, Bill Lam, Chihiro Yamasaki, Remi Sugahara, Meng Li, Yasuhito Tanaka, T. Jake Liang, Chise Tateno, Takeshi Saito

**Affiliations:** 1 Division of Gastrointestinal and Liver Diseases, Department of Medicine, Keck School of Medicine, University of Southern California (USC), Los Angeles, California, USA.; 2 PhoenixBio, Co., Ltd., Higashi-Hiroshima, Hiroshima, Japan.; 3 Bioinformatics Service, Norris Medical Library, USC, Los Angeles, California, USA.; 4 Department of Gastroenterology and Hepatology, Faculty of Life Sciences, Kumamoto University, Kumamoto, Kumamoto, Japan.; 5 Liver Diseases Branch, National Institute of Diabetes and Digestive and Kidney Diseases (NIDDK), NIH, Bethesda, Maryland, USA.; 6 Department of Molecular Microbiology & Immunology,; 7 Department of Pathology, and; 8 USC Research Center for Liver Diseases, Keck School of Medicine, USC, Los Angeles, California, USA.

**Keywords:** Hepatology, Virology, Cytokines, Hepatitis, Innate immunity

## Abstract

Hepatitis delta virus (HDV), a satellite virus of HBV, is regarded as the most severe type of hepatitis virus because of the substantial morbidity and mortality. The IFN system is the first line of defense against viral infections and an essential element of antiviral immunity; however, the role of the hepatic IFN system in controlling HBV-HDV infection remains poorly understood. Herein, we showed that HDV infection of human hepatocytes induced a potent and persistent activation of the IFN system whereas HBV was inert in triggering hepatic antiviral response. Moreover, we demonstrated that HDV-induced constitutive activation of the hepatic IFN system resulted in a potent suppression of HBV while modestly inhibiting HDV. Thus, these pathogens are equipped with distinctive immunogenicity and varying sensitivity to the antiviral effectors of IFN, leading to the establishment of a paradoxical mode of viral interference wherein HDV, the superinfectant, outcompetes HBV, the primary pathogen. Furthermore, our study revealed that HDV-induced constitutive IFN system activation led to a state of IFN refractoriness, rendering therapeutic IFNs ineffective. The present study provides potentially novel insights into the role of the hepatic IFN system in regulating HBV-HDV infection dynamics and its therapeutic implications through elucidating the molecular basis underlying the inefficacy of IFN-based antiviral strategies against HBV-HDV infection.

## Introduction

Hepatitis delta virus (HDV), a satellite virus of HBV, requires hepatitis B surface antigen (HBsAg) for the virion assembly; hence, its life cycle is reliant on the concurrence of HBV infection (1, 2). Currently, at least 20 million people, which is approximately 10% of HBV carriers, are superinfected with HDV worldwide (3–5). While hepatic necroinflammation with chronic HBV infection alone sufficiently leads to the development of end-stage liver diseases, HDV superinfection greatly accelerates the progression of liver disease (6–9). Moreover, HBV-HDV coinfection, or concurrent acute infection, substantially increases the risk of fulminant hepatitis compared with that with acute HBV mono-infection (10). Based on these clinical presentations, HBV-HDV infection has been regarded as the most serious form of viral hepatitis. However, the molecular basis for the profound pathogenesis remains elusive, and there exist no definitive therapeutics, which would entail deciphering the dynamics of virus-host interaction as well as virus-virus interaction.

Viral interference, alternatively referred to as superinfection resistance, has been demonstrated in a multitude of pathogens, wherein a cell infected with the primary pathogen develops resistance to subsequent infection by the superinfectant (11). Notably, HDV superinfection has been shown to interfere with the efficiency of HBV life cycle in vitro, in vivo, and in clinical studies (12–17), indicating that the mode of viral interference in HBV-HDV infection is counterintuitive to the conventional definition; the superinfectant outcompetes the primary pathogen. While the mechanism through which HDV manifests one-way viral interference is unknown, one plausible explanation is that each pathogen possesses a distinctive capacity to elicit and respond to the hepatic antiviral response. HBV infection of hepatocytes has been shown to induce a negligible degree of the IFN system activation (18, 19). On the other hand, albeit controversial, HDV infection involves the activation of the IFN system (20, 21); however, the magnitude and the biological significance of this have not been established to our knowledge.

In general, a potent activation of antiviral response ought to be deleterious to the pathogen; however, one of the IFN-stimulated genes (ISGs), adenosine deaminase acting on RNA 1 (ADAR1), is indispensable for the establishment and maintenance of HDV life cycle (22–24). ADAR1 introduces a site-specific point mutation within the anti-genome, allowing the production of 2 viral proteins from a single open reading frame, the small and large hepatitis delta antigens (HDAgs), which are required for balancing the efficiency of viral genome replication and virion assembly, respectively. Thus, it is plausible that the IFN system acts as a proviral host factor for HDV and that its activation may represent a viral immune exploitation strategy while coincidentally repressing HBV, thereby revealing the paradoxical mode of viral interference.

However, the perceived concept of HBV-HDV interaction mediated through hepatic innate antiviral immunity has been inadequately explored. Herein, we investigated the role and clinical relevance of the hepatic IFN system in the regulation of HBV-HDV infection using highly physiological study models, terminally differentiated human hepatocytes (HH), primary human hepatocytes (PHH), and humanized liver chimeric mice–derived HH (HLCM-HH), for in vitro studies, as well as HLCM for in vivo studies. Our study reveals that HDV infection of hepatocytes resulted in robust and constitutive activation of the IFN system, which potently suppressed HBV infection. Furthermore, the persistent activation of the IFN system in HBV-HDV infection led to a state of IFN tolerance in hepatocytes, which greatly hindered the antiviral efficacy of therapeutic IFN. These findings have significant clinical implications since pegylated IFN (PEG-IFN) is an integral component of antiviral therapy against HBV and HDV. Together, our results suggest that modulating IFN refractoriness could be the key prerequisite for improving the therapeutic outcome of HBV-HDV superinfection.

## Results

### Potent activation of the IFN system in HDV-infected HH.

To develop a comprehensive understanding of the host response to HDV infection, the use of a physiological experimental platform, terminally differentiated HH, is essential; however, this has been also regarded as a research tool with limited versatility because of a rapid loss of genuine characteristics during in vitro culture (25, 26). Consequently, we assessed the longevity of HLCM-HH as well as PHH cultured in vitro with a condition optimized in our previous study (27, 28) (Supplemental Figure 1A; supplemental material available online with this article; https://doi.org/10.1172/jci.insight.162404DS1). We observed that the cell polarity characteristic of terminally differentiated HH, the presence of bile canaliculi between the tight cell-cell contact, were well preserved for at least 28 days, if not longer, according to the immunofluorescence microscopic analysis (IFA) (Supplemental Figure 1A). Moreover, the expression of matured hepatocyte marker genes was maintained at a physiological level in in vitro–cultured HH, which was a stark contrast to their expression in hepatoma cell lines (Supplemental Figure 1B). The expression of sodium taurocholate cotransporting polypeptide (NTCP), the entry receptor of HBV and HDV, in HH was comparable to normal human liver tissue (Supplemental Figure 1, B–D) and was far more abundant than that of NTCP-overexpressing hepatoma cells, which are commonly utilized in HBV and HDV infection studies.

**Figure 1:**
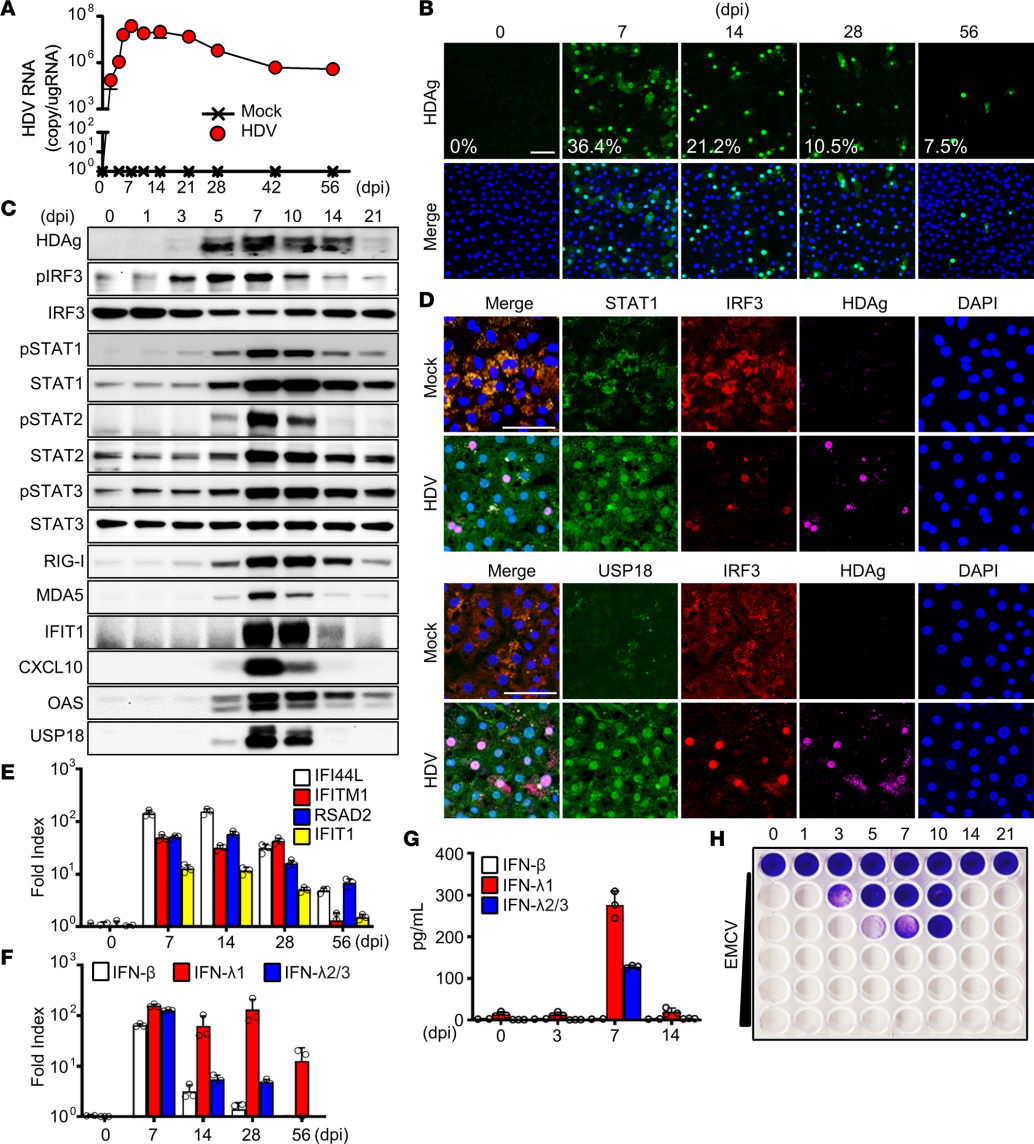
HDV mono-infection of HH and the consequential induction of antiviral response. (**A** and **B**) HLCM-HH mono-infected with HDV (5,000 GEq/cell) for the indicated duration were subjected to the quantification of intracellular HDV RNA via RT-qPCR (**A**) or immunofluorescence microscopic analysis (IFA) for the detection of HDAg (**B**). Green, HDAg; blue, DAPI. Scale bar: 50 μm. The percentage shown in the image indicates the median HDAg-positive foci/total number of cells. Results are shown as mean ± SD of triplicate samples (**A**). GEq, genome equivalents; RT-qPCR, quantitative reverse transcription PCR; dpi, days postinfection. (**C**–**H**) HLCM-HH mono-infected with HDV (5,000 GEq/cell) for the indicated duration were subjected to comparative measurement of protein expression (**C**) or mRNA (**E** and **F**) or ELISA (**G**) for the detection or quantification of indicated molecules. RT-qPCR results represent the relative fold index to the average of the baseline (day 0) normalized by the value of GAPDH. (**D**) HLCM-HH mono-infected with HDV (5,000 GEq/cell) for 7 days followed by immunostaining with indicated molecules. Nuclei were visualized by staining the cells with DAPI (blue). Scale bar: 50 μm. (**H**) Encephalomyocarditis virus (EMCV) cytopathic effect assay. Huh7 cells were first incubated for 24 hours with the culture supernatant of HLCM-HH mono-infected with HDV (5,000 GEq/cell) for 0–21 days, followed by the inoculation with increasing titers of EMCV at 0, 1 × 10^2^, 5 × 10^2^, 1 × 10^3^, 5 × 10^3^, and 1 × 10^4^ PFU/mL for 48 hours prior to crystal violet staining. Displayed data represent one of the biological triplicate experiments (**A**–**H**).

Next, we challenged HLCM-HH with HDV as a mono-infection, which limits viral life cycle to a single round of entry, then evaluated the viral replication kinetics (Figure 1, A and B). HH demonstrated a high susceptibility, and the degree of permissiveness was substantially greater than that of NTCP-overexpressing hepatoma cells (Supplemental Figure 1, E–G). With HLCM-HH, HDV efficiently established a stable infection, and the replication efficiency peaked at 7 days postinfection and gradually declined thereafter; however, it persisted at a high level for at least 56 days despite the inability to produce infectious particles (Figure 1A).

We then evaluated the HH antiviral response against HDV infection and found that interferon regulatory factor 3 (IRF3) was activated in infected cells, evidenced by the nuclear translocation in cells harboring HDAg, followed by the activation of STATs in both HDV-infected and surrounding HDV-naive cells (Figure 1, C and D). We also observed the sustained activation of IRF3 and STATs along with the persistent upregulation of ISGs in HH infected with HDV (Figure 1, C and E). We next characterized the types of IFN produced in HDV-infected HH, revealing that type III IFNs, IFN-λ1 (IL-29) and, to a lesser extent, IFN-λ2/3 (IL-28A/B), were the major factors in ISGs’ induction in neighboring HDV-naive cells (Figure 1, F and G). We determined that the overall antiviral potency of IFNs produced in HDV-infected HH was equivalent to 10 IU/mL of type I and III IFNs (Figure 1H and Supplemental Figure 1H). Last, we verified that HDV infection of PHH also resulted in a robust induction of ISGs and IFNs at levels comparable to those of HLCM-HH (Supplemental Figure 1I). These observations collectively demonstrate that HDV infection of HH induces a potent and prolonged activation of the IFN system.

### HDV coinfection impairs the efficiency of HBV infection via activation of the IFN system.

**Figure 2:**
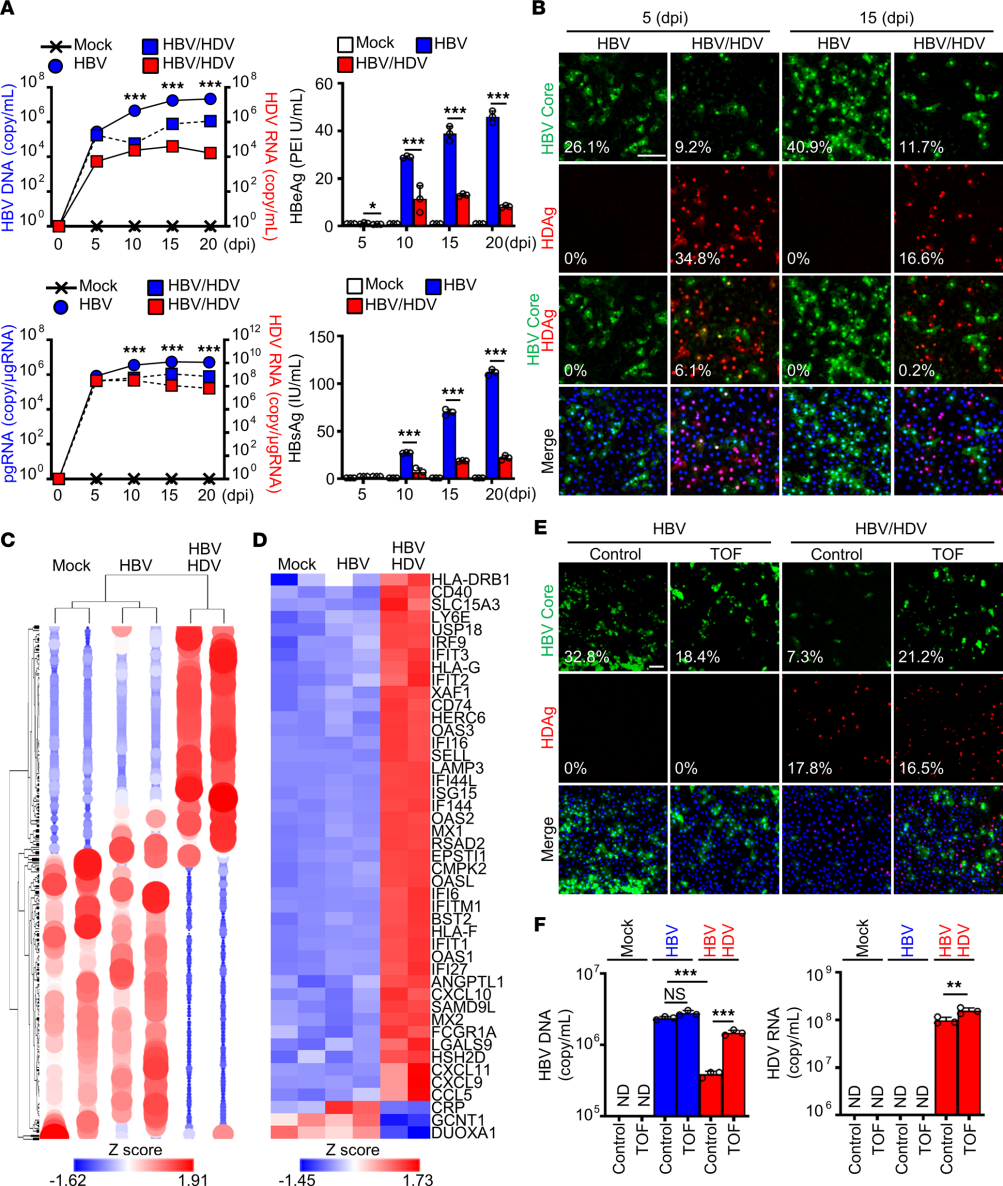
HDV coinfection limits the HBV life cycle through the activation of the IFN system in hepatocytes. (**A**) HLCM-HH were coinfected with HBV (MOI 50) and HDV (5,000 GEq/cell) for indicated durations. The culture supernatants were subjected to the quantification of HBV DNA and HDV RNA (top, left) via RT-qPCR as well as HBeAg (top, right) and HBsAg (bottom, right) with ELISA. Total cellular RNA at each time point was also subjected to the quantification of HBV pgRNA and HDV RNA via RT-qPCR (bottom, left). Results are shown as mean ± SD of triplicate samples. *P < 0.05, ***P < 0.001 were determined by 1-way ANOVA with post hoc Tukey’s test. HBeAg, HBV e antigen; pgRNA, pregenomic RNA; PEI, Paul-Ehrlich international. (**B**) IFA image of HLCM-HH coinfected with HBV and HDV. Green, HBV Core; red, HDAg; blue, DAPI. Scale bar: 50 μm. The percentage shown in the image indicates the median HBV Core, HDAg, or HBV Core-HDAg dual-positive foci/total number of cells. (**C** and **D**) RNA-Seq analysis of HLCM-HH infected with mock virus, HBV, or HBV-HDV coinfection for 15 days. The hierarchical clustering demonstrates the differentially regulated genes (DEGs) (cutoffs used were a *P* value of 0.01 and a fold-change [FC] of 2) (**C**) and the heatmap analysis of ISGs included in the DEGs (**D**). (**E** and **F**) HLCM-HH were either mono-infected with HBV or coinfected with HBV-HDV in the presence of TOF (10 μM) for 10 days followed by IFA of indicated molecules (**E**) or RT-qPCR analysis of HBV DNA and HDV RNA present in the culture supernatant (**F**). Scale bar: 50 μm. The percentage shown in the image indicates the median HBV Core–positive (top) or HDAg-positive (middle) foci/total number of cells. Results are shown as mean ± SD of triplicate samples. **P < 0.01, ***P < 0.001 were determined by 1-way ANOVA with post hoc Tukey’s test. Displayed data represent one of the biological triplicate experiments (**A**, **B**, **E**, and **F**).

HDV achieves a complete and stable life cycle only when the infected cells harbor concurrent HBV infection. Accordingly, in contrast to HDV mono-infection, coinfection of HLCM-HH with HBV and HDV resulted in the production of HDV viral particles, and infection efficiency remained at a high level after reaching a peak 5 days postinfection (Figure 2A). Notably, HDV coinfection significantly reduced the efficiency of HBV genome replication as well as particle production compared with that of HBV mono-infection (Figure 2A). Our IFA revealed that the inhibitory effect of HDV on HBV infection was most pronounced in cells expressing HDAg, whereas the number of HBV-positive foci was also reduced in cells lacking concomitant HDV infection (Figure 2B).

To further comprehend the host response to HDV infection, we conducted an RNA-Seq analysis of HH with HBV-HDV coinfection and HBV mono-infection. HBV-HDV coinfection resulted in a marked transcriptome alteration, whereas HBV mono-infection promoted a minor, perhaps subtle, change (Figure 2C and Supplemental Figure 2A). Accordingly, the bioinformatic analysis revealed that the majority of the differentially expressed genes (DEGs) were found in coinfected cells (97%; 306/314 genes) (Supplemental Figure 2B). Of those 306 DEGs, 205 genes (68%) are known to be significantly up- or downregulated by IFNs, namely IFN-regulated genes (IRGs) (29) (Supplemental Figure 2B). In addition, we found that a significant proportion of upregulated DEGs were ISGs (31%; 43/135 genes) (Figure 2C and Supplemental Figure 2C). On the other hand, HH mono-infected with HBV exhibited only 12 DEGs, of which 5 and 1 are classified into IRGs and ISGs, respectively (Supplemental Figure 2B). Furthermore, the transcriptome analysis of HH mono-infected with HDV indicated that the innate antiviral response triggered in HBV-HDV coinfected cells exclusively was attributed to the immunogenicity of HDV, but not the synergism between HBV and HDV, as all ISGs upregulated in coinfected HH were also induced in cells with HDV mono-infection (Supplemental Figure 2, C and D; Table 1; and Table 2).

Next, HLCM-HH were challenged with HBV mono-infection or HBV-HDV coinfection in the presence of tofacitinib (TOF), a potent inhibitor of Janus kinase (JAK). TOF treatment, at a concentration that sufficiently inhibits IFN-mediated JAK/STAT signaling activation without exhibiting cytotoxicity (Supplemental Figure 2, E–G), greatly relieved HBV from the inhibitory effect of HDV, particularly in cells absent of HDAg expression (Figure 2, E and F). In contrast, TOF treatment exhibited a negligible impact on HBV replication efficiency in the setting of HBV mono-infection. Furthermore, TOF treatment revealed a modest enhancement of HDV replication efficiency (Figure 2F). These findings together suggest that HDV infection elicits a potent innate antiviral response in infected cells, resulting in the establishment of a tissue-wide antiviral state that predominantly inhibits HBV infection.

### HDV superinfection suppresses HBV through the activation of the IFN system.

**Figure 3:**
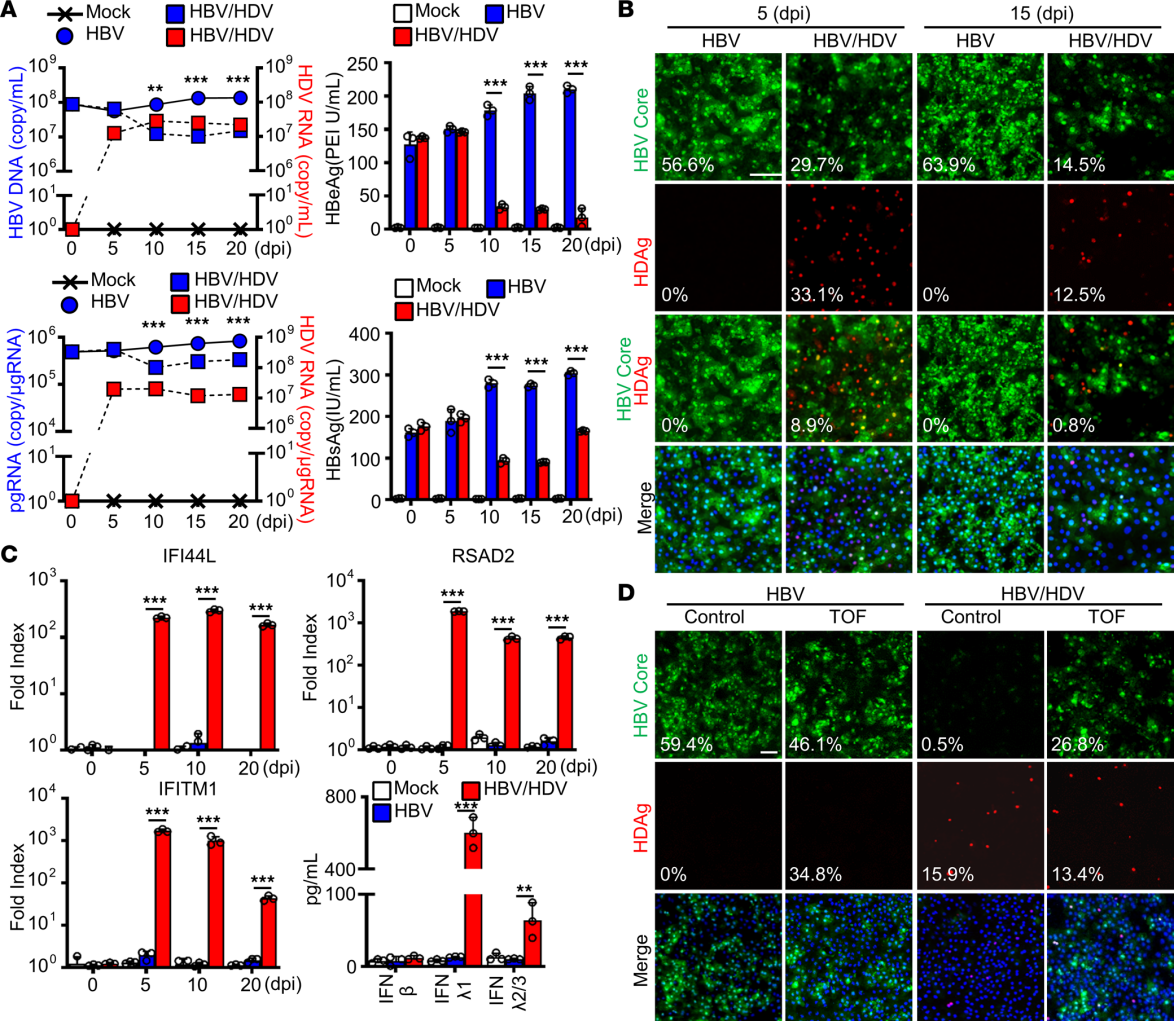
HDV superinfection suppresses HBV through the activation of the IFN system. (**A**) HLCM-HH were first infected with HBV (MOI 50) for 15 days, then superinfected with HDV (5,000 GEq/cell) for indicated durations. The culture supernatants were subjected to the quantification of HBV DNA via RT-qPCR (top, left) or HBeAg (top, right) and HBsAg (bottom, right) with ELISA. Total cellular RNA at each time point was also subjected to the quantification of HBV pgRNA and HDV RNA via RT-qPCR (bottom, left). Results are shown as mean ± SD of triplicate samples. **P < 0.01, ***P < 0.001 were determined by 1-way ANOVA with post hoc Tukey’s test. (**B**) IFA image of HLCM-HH superinfected with HBV and HDV. Green, HBV Core; red, HDAg; blue, DAPI. Scale bar: 50 μm. The percentage shown in the image indicates the median HBV Core, HDAg, or HBV Core-HDAg dual-positive foci/total number of cells. (**C**) Cell lysate and culture supernatant of HLCM-HH with either HBV mono-infection or HBV-HDV superinfection were subjected to the assessment of relative expression changes of indicated ISGs via RT-qPCR and the quantification of indicated IFNs via ELISA. Results are shown as mean ± SD of triplicate samples. **P < 0.01, ***P < 0.001 were determined by 1-way ANOVA with post hoc Tukey’s test. (**D**) HLCM-HH were first infected with HBV (MOI 50) for 15 days followed by superinfection with either mock or HDV in the absence or presence of TOF (10 μM) for 10 days for IFA of indicated molecules. Scale bar: 50 μm. The percentage shown in the image indicates the median HBV Core (top) or HDAg (middle) positive foci/total number of cells. Displayed data represent one of the biological triplicate experiments (**A**–**D**).

Next, we assessed whether HDV superinfection also exhibits an inhibitory effect on the HBV life cycle. To this end, HLCM-HH were first infected with HBV for 15 days, allowing for establishing a robust and stable infection, with almost all cells expressing viral protein and virion production efficiency reaching a plateau (Figure 3, A and B). Similar to our findings with the coinfection model, we observed that HDV superinfection considerably reduced the number of HBV-positive foci, particularly in cells with HDAg expression (Figure 3B). Accordingly, HDV superinfection significantly suppressed the efficiency of HBV genome replication, viral protein expression and secretion, as well as viral particle production (Figure 3, A and B).

Next, we evaluated the status of innate antiviral response over the course of HBV-HDV superinfection. The expression of representative ISGs, selected based on the transcriptome profile of the HBV-HDV–infected cells (Table 1 and Table 2), IFN-induced protein 44-like (IFI44L), IFN-induced transmembrane protein 1 (IFITM1), and radical S-adenosyl methionine domain containing 2 (RSAD2), were all significantly upregulated upon HDV superinfection, and their expression remained at a high level throughout the infection (Figure 3C). Moreover, we verified that IFN-λ1 was the predominant antiviral cytokine produced in HDV-superinfected HH and contributed to the establishment of an antiviral state in neighboring cells (Figure 3C).

We also found that the inhibition of IFN/JAK/STAT signaling by TOF treatment greatly alleviated the inhibitory effect of HDV superinfection on HBV infection efficiency, particularly in cells lacking HDAg expression (Figure 3D). The potency of IFNs produced from HDV-infected cells was further inferred by the enhancement of HBV genome replication efficiency to the level comparable to HBV mono-infection in the presence of TOF (Supplemental Figure 3A). The replication efficiency of HDV, on the other hand, was moderately enhanced with TOF treatment (Supplemental Figure 3B). These findings highlight the potency of HDV-mediated activation of the hepatic IFN system in limiting HBV infection, despite being significantly more stable and robust in the setting of superinfection than that in coinfection. Last, to further substantiate the significance of HDV-mediated activation of the hepatic IFN system, we evaluated the efficiency of HBV infection in HH with preexisting HDV infection, an HBV superinfection model, although this mode of infection is exceedingly improbable in real life. As expected, the repression of HBV replication efficiency was more pronounced in this model (Supplemental Figure 3C) as HH had established antiviral state prior to the establishment of HBV life cycle.

### HDV infection induces the state of IFN refractoriness in hepatocytes.

**Figure 4:**
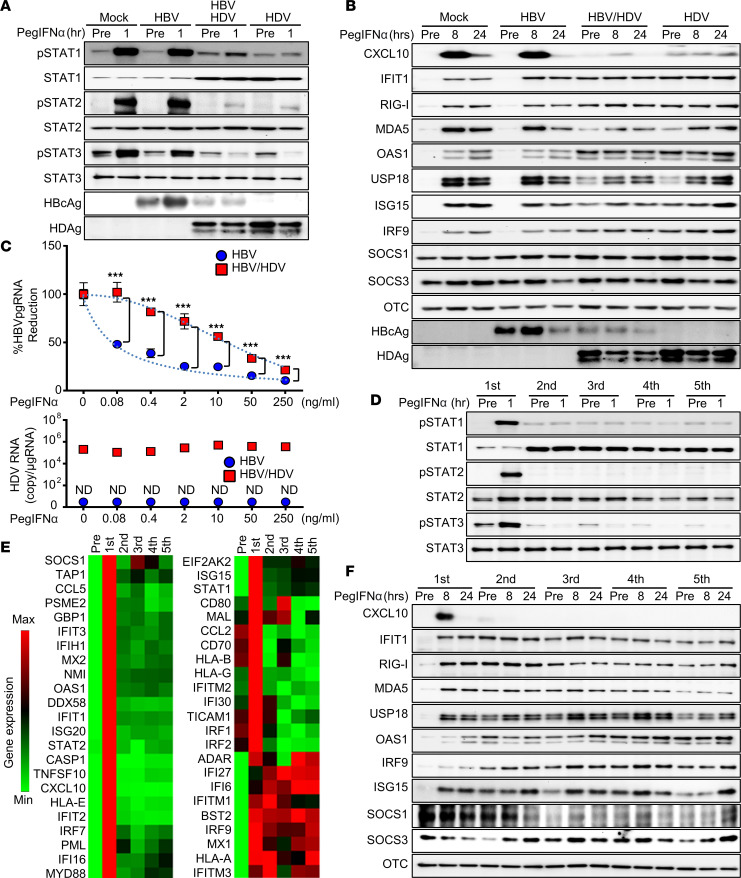
HDV infection induces IFN refractoriness in hepatocytes. (**A** and **B**) HLCM-HH mono-infected with HBV, superinfected with HBV-HDV for 10 days, or mono-infected with HDV for 10 days followed by PEG–IFN-α-2a (10 ng/mL) treatment for indicated hours and the cell lysates subjected to immunoblotting analysis for detection of indicated molecules. (**C**) HLCM-HH mono-infected with HBV or superinfected with HBV-HDV for 10 days were treated with PEG–IFN-α-2a at indicated concentrations for 10 days, and total cell lysates were subjected to quantification of HBV pgRNA (top) and HDV RNA (bottom). Results are shown as mean ± SD of triplicate samples. ***P < 0.001 determined by unpaired 2-tailed Student’s *t* test. (**D**–**F**) HLCM-HH were treated with PEG–IFN-α-2a (10 ng/mL) for 5 days per cycle, up to 5 times. Total cell lysates were harvested at the indicated time points for immunoblotting analysis for detection of respective proteins (**D** and **F**) or 8 hours after each treatment for RT-qPCR array analysis of ISGs (**E**). Results are shown as mean ± SD of triplicate samples. Displayed data represent one of the biological triplicate experiments (**A**–**F**).

The potent activation of the IFN system is expected to be detrimental for viral pathogens; meanwhile, the induction of ADAR1, one of the ISGs, is indispensable for the HDV life cycle. Consequently, we postulated that the HDV life cycle is maintained on an exquisite balance between its ability to potently induce innate antiviral responses and its high resistance to the antiviral action of the IFN system. To test this hypothesis, we first assessed the impact of HDV infection on HH response to IFN treatment. The degree of JAK/STAT signaling activation by IFN treatment and the subsequent ISG induction were significantly reduced in HH infected with HDV and HBV-HDV, whereas the IFN response in HBV mono-infected HH was comparable to that of control cells (Figure 4, A and B, and Supplemental Figure 4A). We also observed that the activation status of JAK/STAT signaling and ISGs’ expression at the baseline were higher in HDV-infected HH (Figure 4, A and B, and Supplemental Figure 4A). Moreover, these upregulated ISGs in HDV-infected cells included well-accepted negative regulators of IFN signaling, such as USP18 and ISG15. Collectively, these observations point to a possibility that the constitutive activation of the IFN system by HDV leads to an equilibration between high basal levels of ISGs’ expression and irresponsiveness to exogenous IFN.

To further delineate the relevance of HDV-induced hepatic IFN refractoriness, we tested the effectiveness of therapeutic IFNs in the suppression of HBV as well as HDV. Notably, the efficacy of PEG–IFN-α for the suppression of HBV was substantially blunted in HBV-HDV infection as compared with HBV mono-infection, especially at lower concentrations, but remained statistically significant at therapeutic doses (Figure 4C). In addition, we found that IFN therapy, even at supratherapeutic doses, failed to inhibit HDV genome replication (Figure 4C). We postulated that the constitutive activation of the IFN system by HDV infection is the predominant attribute for IFN refractoriness rather than viral components subverting IFN production or response in infected HH. Indeed, the ectopic expression of HDV proteins, large HDAg (L-HDAg) and small HDAg (S-HDAg), in HH had no effect on the cellular response to IFN or the ability to produce IFNs (Supplemental Figure 4, C–E).

To further test our hypothesis, we examined whether recurrent IFN treatment establishes IFN refractoriness in HH. Upon initial exposure to IFN-α, HH exhibited a robust activation of JAK/STAT signaling and consequent induction of ISGs; however, subsequent IFN doses neither augmented nor sustained the responsiveness, resulting in the overall attenuation of ISGs’ expression, with the exception of a small subset of genes (Figure 4, D–F). In addition, following exposure to consecutive doses, the expression abundance of the majority of, but not all, ISGs returned to a level comparable to or lower than the baseline (Figure 4E). These findings collectively suggest that constitutive activation of the IFN system as a result of the host response to HDV infection leads to IFN refractoriness.

### HDV infection leads to the state of IFN refractoriness in vivo.

To further define the significance and clinical implication of HDV-induced hepatic IFN refractoriness, we conducted an in vivo study with the uPA-SCID-based HLCM system. HLCM represent a suitable small animal model for in vivo study of HBV and HDV infection since the host tropisms of these pathogens are confined to humans and chimpanzees. In addition, the absence of the adaptive immune system in the SCID-based system provides an advantage for elucidating the role of the innate immune system (30).

**Figure 5:**
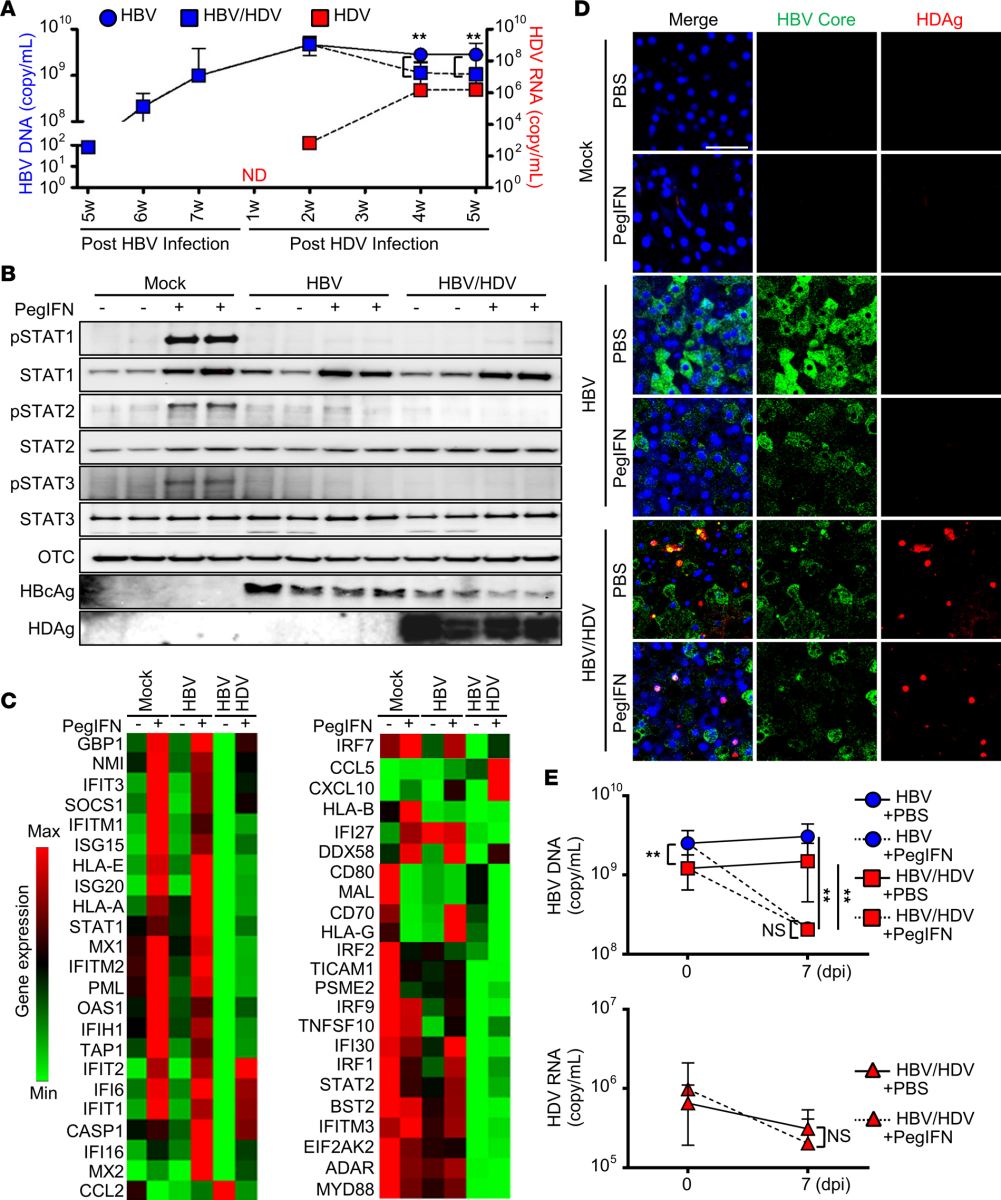
HDV infection induces the state of IFN refractoriness in hepatocytes in vivo. (**A**–**E**) HLCM (*n* = 40) were first infected with either mock (*n* = 12) or HBV (*n* = 28) for 7 weeks. HLCM infected with HBV (*n* = 28) were then superinfected with mock (*n* = 12; HBV mono-infection) or HDV (*n* = 16; HBV-HDV superinfection) for an additional 5 weeks. Blood samples of each animal at indicated time points were subjected to the quantification of HBV DNA and HDV RNA via RT-qPCR (**A**). (**B**–**E**) HLCM infected with mock, HBV, or HBV-HDV were administered with PBS or PEG–IFN-α-2a, which were killed for sampling as outlined in Supplemental Figure 5 for comparative analysis. Representative results of immunoblotting analysis (**B**) and RT-qPCR array analysis (**C**) of the liver tissue, harvested 24 hours after PBS or PEG–IFN-α-2a administration. Representative IFA image of the liver tissue (**D**) as well as RT-qPCR analysis of blood samples (**E**) harvested 7 days after PBS or PEG–IFN-α-2a administration. **P < 0.05 determined by unpaired 2-tailed Student’s *t* test. Error bars, SD. Scale bar: 50 μm.

HLCM were first inoculated with either mock virus or HBV for 7 weeks to allow for achieving a stable HBV mono-infection (Figure 5A and Supplemental Figure 5). Then, HBV-infected HLCM were challenged with either mock or HDV infection to establish HBV mono-infection or HBV-HDV superinfection. The serial blood test demonstrated that the serum HDV RNA titer gradually increased, and the replication efficiency reached a plateau 4 weeks postinoculation. Consistent with the results of our in vitro studies (Figures 2 and 3), both serum HBV DNA titer and HBV viral protein abundance in the liver tissue were lower in HLCM with HDV superinfection compared with those of HBV mono-infected animals (Figure 5, A, B, and D).

In order to assess whether HDV superinfection impairs the responsiveness to therapeutic IFN in vivo, HLCM with mock, HBV, and HBV-HDV infection were administered with either PBS or PEG–IFN-α, and then the liver tissue was examined for the activation status of JAK/STAT signaling (Figure 5, B–D, and Supplemental Figure 5). In accordance with our in vitro study results, the activation of JAK/STAT signaling in response to the therapeutic dose of PEG–IFN-α (30 μg/kg) was substantially compromised in HDV-superinfected animals compared with that of the control animals (Figure 5, B and C). Of important note, the magnitude of STAT activation was also significantly weakened in HBV mono-infected animals (Figure 5C), which was in stark contrast to what we observed in vitro (Figure 4B). The diminishing level of IFN signaling activation in the superinfected animals was associated with the overall impairment of ISGs’ induction (Figure 5, B and C). Moreover, the expression abundance of most ISGs at the baseline in HBV-HDV–infected animals was even lower than those of uninfected as well as HBV-monoinfected animals, which well resembled the pattern seen in HH following long-term exposure to therapeutic IFN (Figure 4E). In contrast, despite reduced magnitude of STATs’ activation, HBV mono-infected animals exhibited relatively preserved responsiveness to therapeutic IFN to the induction of ISGs (Figure 5C).

Next, we evaluated the antiviral efficacy of therapeutic IFN against HBV and HDV in vivo. We observed a greater degree of serum HBV DNA titer reduction in HBV mono-infected animals than that of HDV-superinfected animals; however, the difference was not statistically significant (Figure 5E). In accordance with our in vitro studies, the serum HDV RNA titer remained unchanged in HLCM administered therapeutic PEG–IFN-α (Figure 5E), revealing a lack of antiviral effectiveness against HDV infection in vivo.

## Discussion

The present study investigated the significance and therapeutic implications of hepatic antiviral innate immunity in the regulation of HBV-HDV infection using a 2D HH culture system and uPA-SCID–based HLCM system. These experimental platforms retain the genuine characteristics of HH and are thus considered far more physiologically relevant than other study tools, such as hepatoma cell lines and human NTCP-transgenic mice, which have only a marginal similarity to the original primary cells and suffer from interspecies differences, respectively (30).

Our work demonstrated that HDV infection of HH results in a robust induction of ISGs, the magnitude of which is substantial enough to suppress the replication efficiency of HBV in coinfected or superinfected cells as well as in superinfected animals. Moreover, IFNs, primarily IFN-λ1, secreted from HDV-infected HH facilitate the establishment of an antiviral state in neighboring cells in a paracrine manner, resulting in a tissue-wide suppression of HBV even in cells without concomitant HDV infection. In contrast, HBV infection of HH induces a negligible level of host response, as evidenced by a minimal alteration in the transcriptome landscape, thereby reinforcing the notion that HBV is a stealth virus (18). This concept, however, might only be valid to in vitro studies, as our in vivo study demonstrated perturbation of JAK/STAT signaling activation in HBV-infected HLCM, which may, at least to a certain extent, explain the discordance between in vitro and in vivo study results. Our study also revealed that, compared with HBV, HDV possesses a substantial level of resistance to the antiviral effects of IFN. These observations together suggest that the one-way suppression of HBV by HDV is attributed to each pathogen’s distinct immunogenicity in activating the IFN system and differential susceptibility to ISGs. Notably, this phenomenon stands in contrast to the conventional concept of viral interference, in which a cell infected with a primary virus acquires resistance to subsequent infection of a superinfectant virus (11, 31). The paradoxical mode of viral interference between HBV and HDV is deemed clinically relevant as the serum HBV DNA titer is considerably lower in individuals with HDV superinfection than those with HBV mono-infection (14–17). Our study results, for the first time to our knowledge, establish a compelling explanation on how the superinfectant, HDV, achieves the unidirectional suppression of the primary pathogen, HBV, through the activation of the hepatic IFN system.

Given that the IFN system is the foundation of the antiviral defense program, its activation ought to be detrimental to viral pathogens; however, such a principle might not be applicable to HDV infection. This notion is underscored by our observation that the suppression of IFN signaling with TOF treatment results in a subtle enhancement of HDV replication efficiency, which is congruent with the negligible inhibitory effects of therapeutic IFN on HDV replication seen in our in vitro and in vivo studies. Along with the fact that the HDV life cycle is dependent on one of the ISGs, ADAR1, our findings suggest that HDV not only has a high resistance to the antiviral properties of ISGs but also leverages the innate antiviral defense program to sustain its own viral life cycle.

Of important note, our work also suggests that the persistent activation of the IFN system in HDV infection leads to a tissue-wide state of IFN refractoriness. The magnitude of ISG induction is highest upon the initial exposure and gradually decreases toward the baseline over the course of infection, during which time HH loses its IFN responsiveness. We postulate that this phenomenon is mediated through the HH response to a constitutively active IFN system rather than by viral factors. This hypothesis is well supported by our observations with HH treated with therapeutic IFN. ISGs’ expression abundance increases substantially in response to the initial dosage, as it does with HDV infection; nevertheless, HH develop refractoriness to subsequent doses. As a result, the overall expression abundance of ISGs decreases over time and approaches a level comparable to that in HH unexposed to IFN.

IFN refractoriness, alternatively IFN tolerance, is a phenomenon first reported in 1967 (32); the cells produced or sensitized to IFN lose their potential to reproduce and/or respond to IFN upon restimulation, prohibiting auto-amplification of IFN system activation following acute viral infection. In general, this mechanism is considered a host-protective process, preventing excessive immune activation, collateral tissue injury, and the development of autoimmune disorders (33). Consequently, persistent activation of the IFN system results in chronic necroinflammation of the affected tissue and ultimately organ failure as seen in chronic hepatitis C virus infection, where excessive hepatic ISG expression is linked with worse clinical outcomes (34, 35). Consequently, our observation, the persistent and robust activation of the IFN system by HDV, might explain, at least in part, the accelerated progression of liver fibrosis and the increased risk of developing end-stage liver diseases seen in patients with chronic HBV-HDV infection.

To mitigate the disease burden of HDV infection, the establishment of effective antiviral strategies is imperative. Type I IFN has been employed as the mainstay antiviral therapy for HDV infection; nevertheless, the treatment outcome data have all pointed to the lack of effectiveness (36–39). We postulate, based on our observations, that the ineffectiveness is largely owing to the hepatic IFN refractoriness established through the host response to HDV infection and the high resistance of HDV to the antiviral properties of ISGs. Several novel antiviral agents against HDV have been developed and are currently being evaluated in clinical trials, including lonafarnib and bulevirtide, which inhibit virion assembly and viral entry, respectively (40–42). While the effectiveness of these compounds remains undetermined because of the lack of long-term posttreatment follow-up data, these drugs are proposed to be used in combination with IFN (43); hence, the high resistance to IFN along with HDV-induced hepatic IFN tolerance are expected to be significant obstacles.

Anti-HBV therapeutics that lead to the functional cure, defined as the loss of HBsAg, could be an alternative approach for HDV eradication. While current anti-HBV mainstays, nucleoside/nucleotide analogs, have limited efficacy in inducing functional cure, more than 30 novel compounds are under development (44). The clinical trial findings are encouraging for some, such as REP 2139 (nucleic acid polymer-based HBsAg release inhibitor) and GS-9620 (TLR-7 agonist), in terms of the potential of achieving functional cure (45, 46); however, these drugs are also proposed to be administered in combination with IFN, or the mechanism of action mimics the antiviral action of IFN. Therefore, the prospective utility of these drugs, as a means of eradicating HDV, remains uncertain, given that dual-infected populations are predicted to have reduced responsiveness to IFN treatment.

The present work provides multiple insights into the role of the hepatic IFN system in the regulation of HBV-HDV infection dynamics. Our observation also suggests the therapeutic implication of the hepatic IFN refractoriness inflicted by the constitutive activation of hepatic innate immunity in persistent HDV infection. Accordingly, the establishment of a strategy that temporarily disrupts the state of IFN refractoriness prior to the initiation of antiviral therapy could be a measure for improving treatment outcomes for patients with HBV-HDV dual infection. These concepts supported by our work, however, have to be validated further with a clinically relevant, long-term IFN treatment schedule to determine whether HDV infection negatively impacts the efficacy of IFN-based anti-HBV and anti-HDV therapy. Last, given the IFN system plays a significant role in the activation of T and B cells, future study with an experimental system equipped with the adaptive immune system, such as dual-humanized mice (47), is crucial for determining the clinical implications of our proposed concept.

## Methods

### Cells and tissues.

Cryopreserved PHH from 3 donors were obtained as cryopreserved vials (BioIVT). HLCM-HH were isolated from the livers of uPA-SCID–based HLCM with a standard 2-step collagenase perfusion method as previously described (27). HLCM used as the source of HLCM-HH were established with PHH obtained from 2 donors (27). PHH and HLCM-HH were seeded on a type I collagen–coated cell culture dish, BioCoat (Corning), at the cell density of 2.1 × 10^5^ cells/cm^2^ and cultured with DMEM-based media as described previously (27). Huh7 (JCRB Cell Bank), Huh7.5 (a gift from T. Wakita, National Institute of Infectious Diseases, Tokyo, Japan), HepG2 cells overexpressing NTCP (a gift from Ju-Tao Guo, Blumberg Institute, Doylestown, Pennsylvania, USA), and their respective control cells were maintained with previously described conditions (48). Normal human liver tissue from 3 different donors was obtained as described previously (49).

### Animals.

cDNA-uPA^+/–^/SCID (uPA^+/WT^: B6;129SvEv-Plau, SCID: C.B-17/Icr-scid/scid Jcl) strain (PhoenixBio) was used for the production of HLCM as described previously (50).

### Viruses.

HDV was propagated using Huh 7.5 cells cotransfected with pT7HB2.7, a plasmid encoding a subgenomic HBV fragment (genotype D, 2.7 kb) (a gift from Camille Sureau, Institut National de la Transfusion Sanguine, Paris, France) (51), and pSVLD3 (genotype 1, American Type Culture Collection), the culture supernatant of which was harvested 9 days after transfection and stored at –80°C. HBV inoculum was propagated either with HLCM (genotype A or C) or in vitro (genotype D) as described previously (52, 53). HBV and HDV infections to PHH or HLCM-HHs were carried out at indicated titers in the presence of 4% polyethylene glycol 8000 (Promega) for 24 hours as previously described (54). In vivo HBV and/or HDV infections of HLCM (16–26 weeks old) were performed via retro-orbital inoculation at the specified titer. Sendai virus (Cantell strain) (Charles River Laboratories) infection was carried out at 100 hemagglutination units/mL in serum-free DMEM for 1 hour at 37°C. Lentivirus particles were propagated as described previously (55). Cytopathic effect assay was performed with Huh7 cells treated with testing samples or IFN standards with indicated concentration for 24 hours followed by incubation in the presence of serially diluted EMCV for an additional 48 hours. At endpoint, cells were stained with crystal violet to visualize viable cells.

### Nucleic acid.

pSVLD1, a plasmid harboring unit length of HDV genome, was generated by EcoRI digestion of pSVLD3 followed by self-ligation with T4 DNA ligase (New England Biolabs). HDV RNA reference standard was prepared via in vitro transcription of BamHI-linearized pSVLD1 using MEGAscript SP6 Kit (Thermo Fisher Scientific). The open reading frames of L-HDAg and S-HDAg were PCR-amplified from pSVLD3 and subcloned into pCDH-CMV-MCS-EF1-Puro (System Bioscience) for the lentiviral particle production. DNA transfection was carried out using TransIT-LT1 Transfection Reagent (Mirus Bio). Viral genomes were extracted from cell culture supernatant and cell lysate using QIAamp MinElute Virus Spin Kit (QIAGEN) and Quick-DNA/RNA Viral Kits (Zymo Research), respectively. The quantities of viral genome were determined via probe-based RT-qPCR using TaqMan Fast Virus 1-Step Master Mix (Thermo Fisher Scientific) with the following probes and primer sets: HDV: probe: 5′-6-FAM-AGGCGCTTCGAGCGGTAGGAGTAAGA-QSY-3′, forward primer: 5′-GGACCCCTTCAGCGAACA-3′, and reverse primer: 5′-CCTAGCATCTCCTCCTATCGCTAT-3′. HBV DNA: probe: 5′-FAM-CAGAGTCTAGACTCGTGGTGGACTTC-TAMRA-3′ forward primer: 5′-CACATCAGGATTCCTAGGACC-3′, and reverse primer: 5′-AGGTTGGTGAGTGATTGGAG-3′, HBV pgRNA: probe: 5′-6-FAM-AGGCAGGTCCCCTAGAAGAAGAACTCC-QSY-3′, forward primer: 5′-GGAGTGTGGATTCGCACTCCT-3′, and reverse primer: 5′-AGATTGAGATCTTCTGCGAC-3′. HDV and HBV standards for the calibration curve were prepared using a 10-fold dilution series of in vitro–transcribed unit length HDV genome and serially diluted pHBV1.3, respectively, which was linear over 8 orders of magnitude and sensitive down to 10 copies of RNA or DNA transcript. Genome copies were expressed as log_10_ genome copies of RNA or DNA per gram of total cellular RNA, per milliliter of cell culture supernatant, or serum, using a standard curve. The gene expression analyses were carried out via 2-step RT-qPCR using PowerUp SYBR Green Master Mix (Thermo Fisher Scientific), for which total cellular RNA was extracted with Quick-RNA miniprep kit (Zymo Research) followed by cDNA synthesis using qScript cDNA SuperMix (Quantabio). Otherwise noted, RT-qPCR results were presented as relative fold index to the average of the baseline or control condition normalized by the value of GAPDH. The primer sequences for gene expression studies are summarized in Supplemental Table 1. Quantitative PCR array analysis was conducted using RT^2^ Profiler PCR Array (384-well format) Human Type 1 Interferon Response (QIAGEN).

### Chemicals and cytokines.

We used TOF (pan-JAK inhibitor) (MilliporeSigma), PEG–IFN-α (Chugai Pharmaceutical), and recombinant hIFN-β, hIFN-γ, and hIFN-λ (Bio-Techne).

### Protein analysis.

Immunoblotting analyses were carried out via SDS-PAGE of cell lysates prepared in ProPrep (Bulldog-Bio) supplemented with phosphatase inhibitor mixture II (MilliporeSigma), which were then transferred to PVDF membranes followed by blocking with 5% BSA, incubation with primary antibodies targeting indicated proteins, and HRP-conjugated secondary antibodies. The specific signal was detected using SuperSignal West Pico or Femto chemiluminescent substrate (Thermo Fisher Scientific). The antibodies used were as follows: anti-GAPDH (clone 6C5; Santa Cruz Biotechnology), anti-STAT1 (42H3; 9175), anti–phospho-STAT1 (58D6; 9167), anti-STAT2 (D9J7L; 72604), anti–phospho-STAT2 (Tyr690) (D3P2P; 88410), anti-ISG15 (22D2; 2758), anti-USP18 (D4E7; 4813), anti-CXCL10 (D5L5L; 14969), anti–RIG-I (D14G6; 3743), anti-MDA5 (D74E4; 5321), anti-ADAR1 (D7E2M; 14175), anti-IRF3 (D83B9; 4302), anti–phospho-IRF3 (Ser386) (E7J8G; 37829) (Cell Signaling Technology), anti-HBsAg (A10F1), anti-HBV Core antigen (216A) (Cell Marque), anti-ALB (GTX102419), anti-OTC (GTX105140), anti-HDV (GTX 135575 or patient serum; ref. 17) (Genetex), IFIT1 (a gift from Ganes C. Sen, Lerner Research Institute, Cleveland, Ohio, USA), and anti-OAS1 (clone 1.3.3; Kineta). ELISAs for the detection and quantification of following molecules were carried out according to the manufacturers’ protocols: human IFN-β (Bio-Techne), human IL-28A/IFN-λ2, human IL29/IFN-λ1 (RayBiotech), HBV surface antigen, and HBV e antigen (International Immunodiagnostics).

### Microscopic analysis.

The cells plated on 8-chamber slides (ibidi) were fixed with 10% formalin for 10 minutes. The liver tissues from HLCM were fixed overnight at room temperature with 10% formalin followed by sequential buffer exchange with 1× PBS containing 10% and 30% sucrose prior to snap-freezing the tissue embedded in OCT compound at –150°C. The frozen tissues were sliced to a thickness of 5 μm using a microtome-cryostat (Thermo Fisher Scientific), and the tissue section was attached to a microscope slide. The cells or tissues on the microscope slides were then permeabilized with 0.2% Triton X-100 in 1× PBS for 15 minutes at room temperature. Slides were then incubated with blocking buffer (1× PBS containing 5% BSA) for 1 hour prior to incubation with primary antibodies overnight at 4°C and secondary antibodies (Alexa Fluor 488–goat anti-rabbit, Alexa Fluor 647–goat anti-human, or DyLight 550–goat anti-rabbit) (Jackson ImmunoResearch 111-545-144, Jackson ImmunoResearch 109-606-088, and Thermo Fisher Scientific 84541, respectively) for 1 hour at room temperature. The immunostained slides were mounted with DAPI-containing mounting medium (Vector Laboratories). The images were captured using Leica TCS SP8 confocal microscopy system at the Cell and Tissue Imaging Core of the USC Research Center for Liver Diseases and analyzed using a Leica confocal microscope with LAS X software (Leica). The percentage in the microscopic image is the median positivity of the indicated protein-positive foci per 100 cells in 3 independent representative fields.

### Next-generation sequencing and bioinformatics analyses.

Total cellular RNA was first applied to the quality control using Agilent 2100 Bioanalyzer followed by cDNA library synthesis using NEB Next Ultra II RNA Library Prep Kit for Illumina (New England Biolabs). Single-end 75 bp sequencing was carried out with Illumina NextSeq 500. RNA-Seq data were analyzed with Partek Flow version 6. Raw sequencing reads were first trimmed from both ends with a quality score method (bases with a quality score of <20 were trimmed from both ends, and trimmed reads shorter than 25 nucleotides were excluded from downstream analyses). Trimmed reads were then mapped to human genome hg38 using Star version 2.4.1d with default parameter settings and using Gencode v25 annotation as guidance (56). Gencode v25 annotation was used to quantify the aligned reads to genes using Partek’s optimization of the expectation-maximization algorithm method. Genes with fewer than 10 raw reads in all samples were excluded from downstream analysis. Finally, read counts per gene in all samples were normalized using upper quartile normalization (57) and analyzed for differential expression using the Partek gene-specific analysis method. Significantly DEGs were selected using a *P* value of less than 0.01 and an FC of more than 1.5 (or less than –1.5). For hierarchy clustering (HC), genes were standardized before being subjected to unsupervised hierarchical clustering by average linkage clustering with Euclidean distance metric. In HC analysis, both plots’ color and size are representing the *z* score. Principal component analysis (PCA) was performed on normalized read counts, and samples from the same sample group (biological duplicates) are connected and highlighted for better visualization. The ellipsoids were colored by sample group. Both HC and PCA were done using Partek Flow software. The RNA-Seq data have been deposited in the NCBI Gene Expression Omnibus under accession number GSE205567.

### Statistics.

Statistical analysis was performed using Prism version 9 (GraphPad). Significant differences were determined by unpaired 2-tailed Student’s *t* test or 1-way ANOVA with post hoc Tukey’s test for multiple comparisons as appropriate, unless otherwise stated in the figure legend. Data are presented as means ± SD. *P* values of less than 0.05 were considered significant.

### Study approval.

All animal work was performed in accordance with NIH guidelines in conjunction with the protocol approved by the IACUC at the USC and PhoenixBio Co., Ltd. Human liver tissue was obtained under an approved IRB protocol at the USC with donors’ written informed consent.

## Supplementary Material

Supplemental data

## Figures and Tables

**Table 2:**
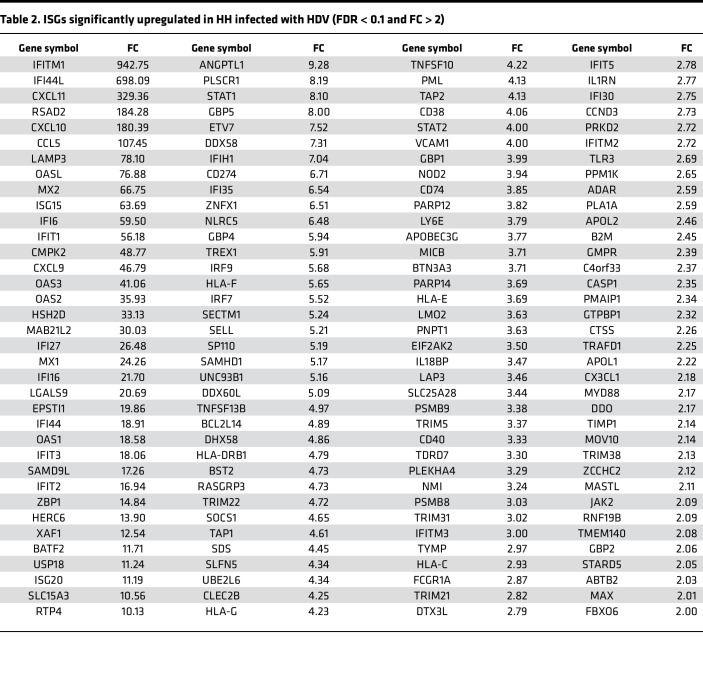
ISGs significantly upregulated in HH infected with HDV (FDR < 0.1 and FC > 2)

**Table 1:**
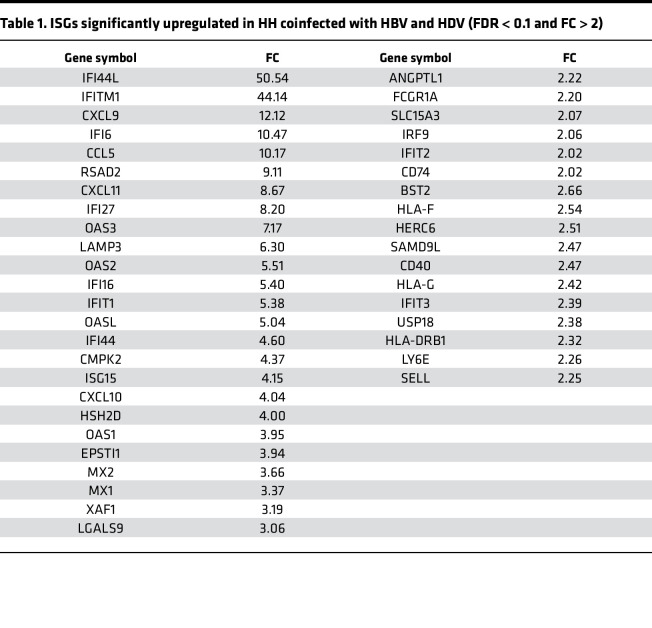
ISGs significantly upregulated in HH coinfected with HBV and HDV (FDR < 0.1 and FC > 2)

## Version 1 05/08/2023

Electronic publication
