# Supplementary material for: Persistent hepatic IFN system activation in HBV-HDV infection determines viral replication dynamics and therapeutic response
Source: JCI Insight. 2023 May 8;8(9):e162404. doi: 10.1172/jci.insight.162404 (PMC10243812; doi:10.1172/jci.insight.162404)
Supplement: Supplemental data [file jciinsight-8-162404-s133.pdf]

Supplemental Figure 1

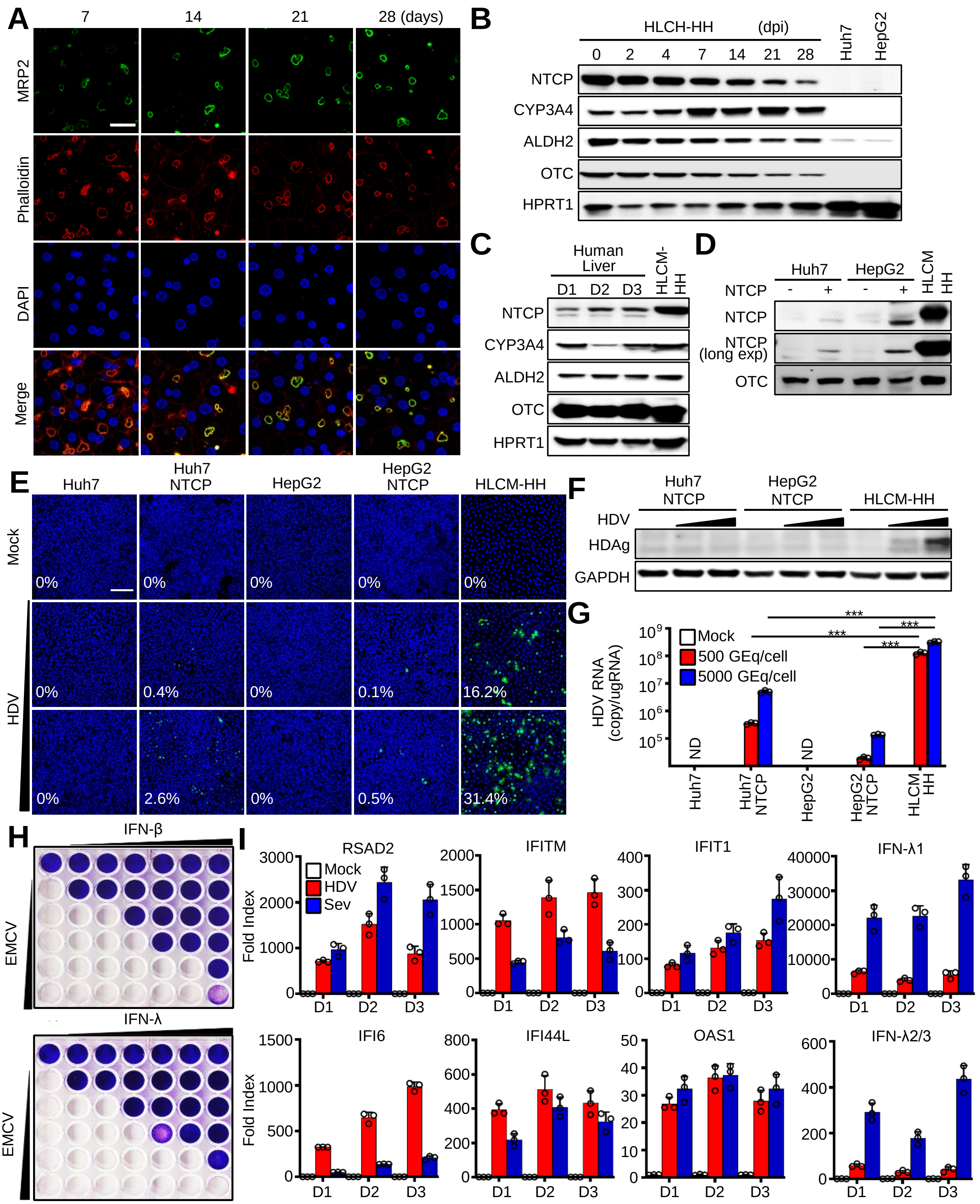

Supplemental Figure 2

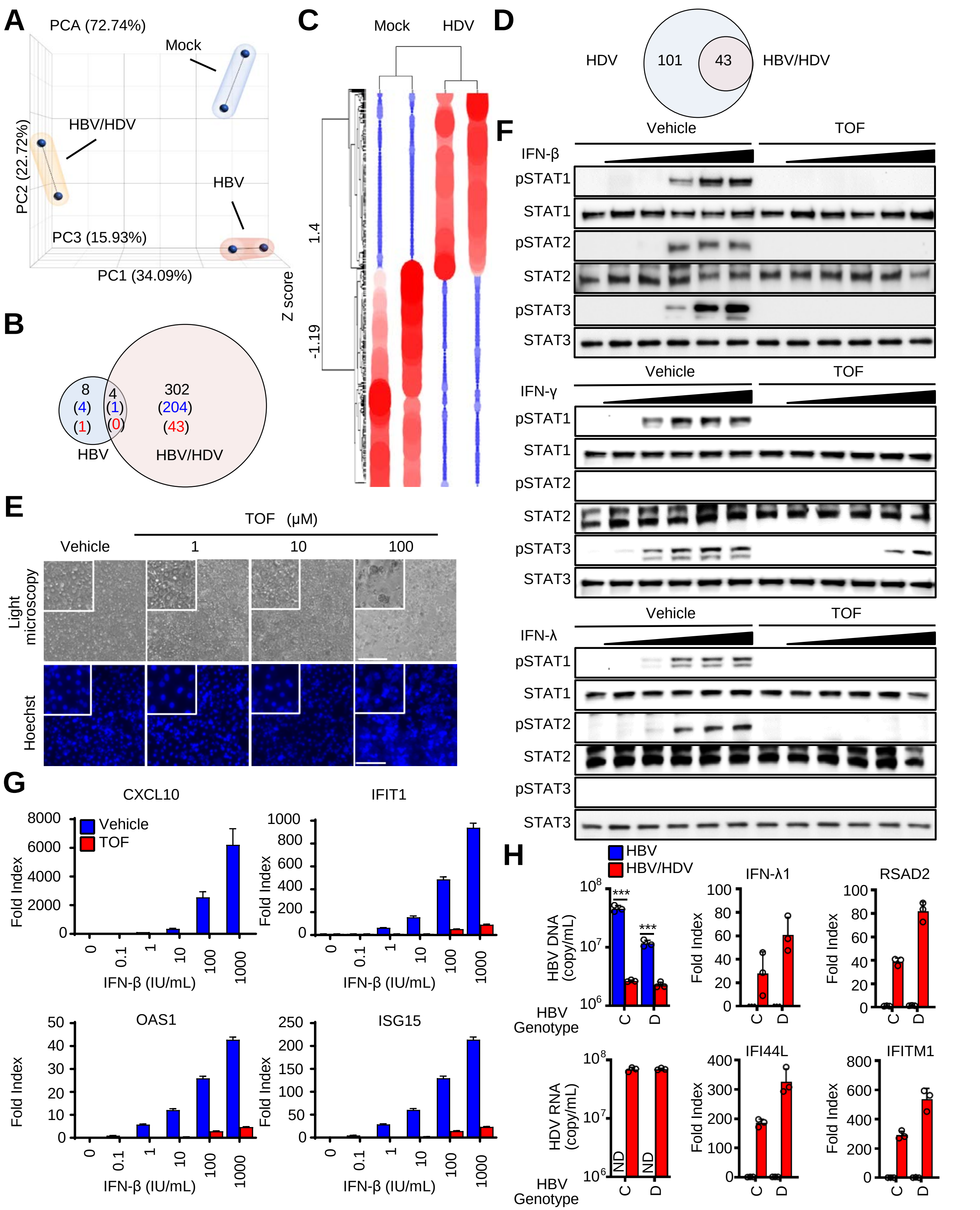

Supplemental Figure 3

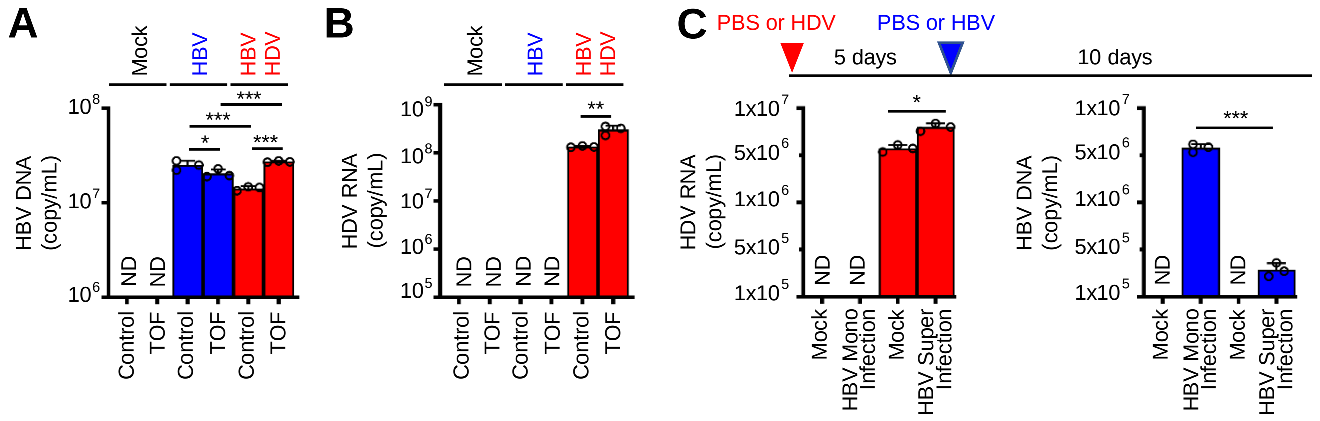

Supplemental Figure 4

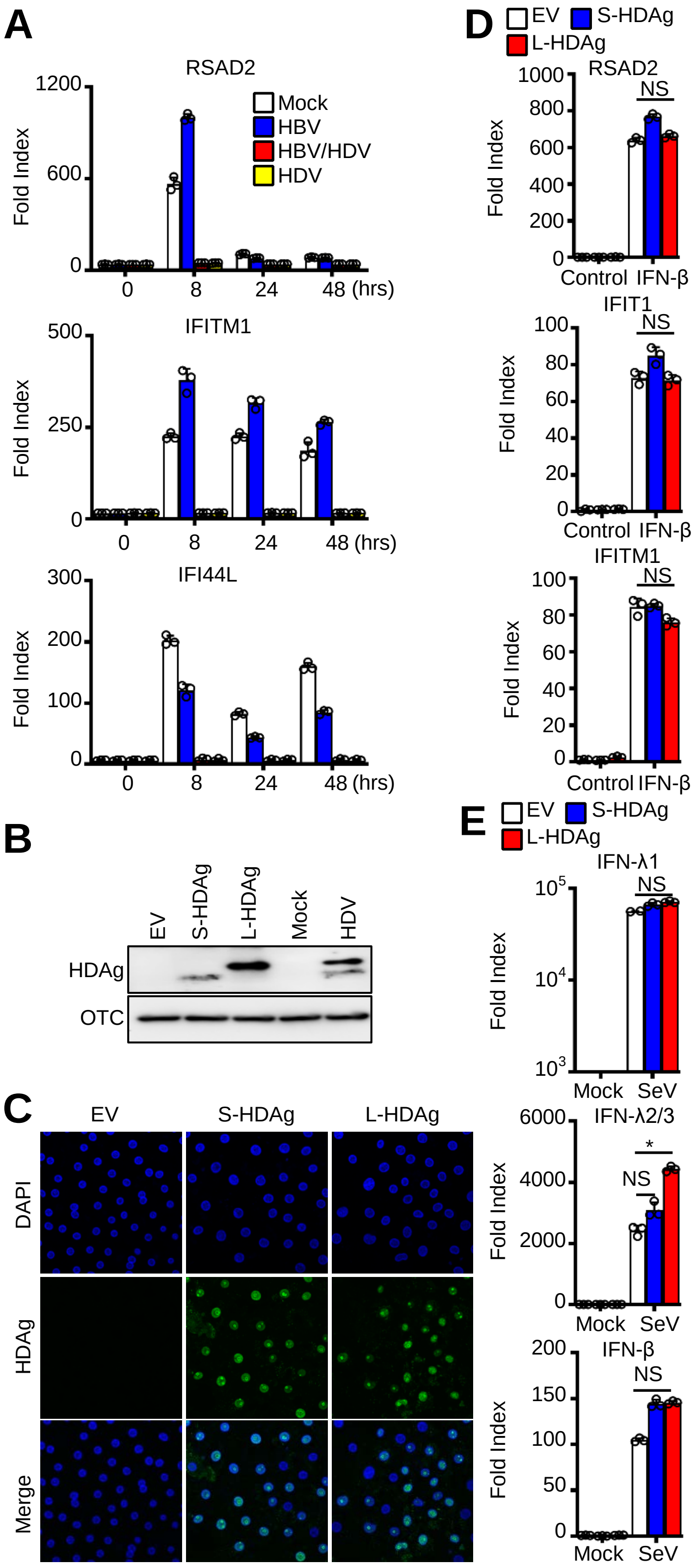

Supplemental Figure 5

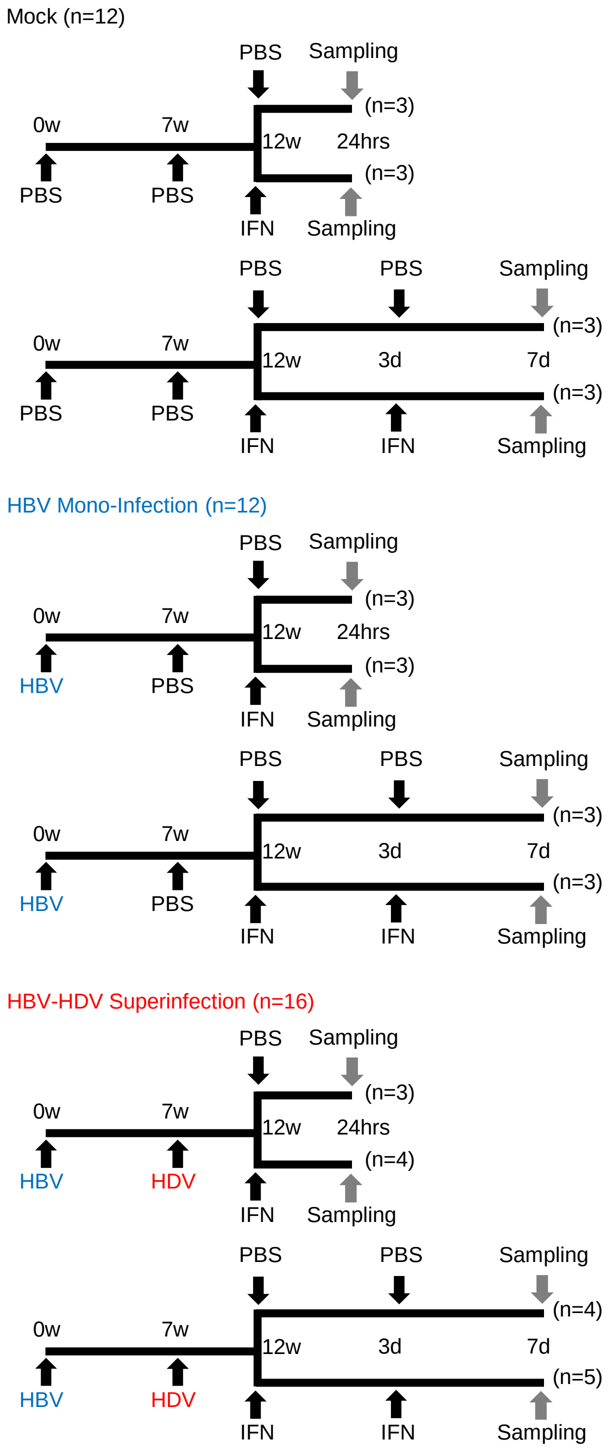

Supplemental Figure Legend

Supplemental Figure 1. Representative images of in vitro cultured HCLM-HH for indicated durations followed by immunofluorescent microscopic analysis (IFA) for the assessment of cellular polarity through the detection of MRP2, the bile canaliculi marker, along with phalloidin staining. A similar result was obtained with PHH from three different donors. Scale bar: 25µm. **B-D.** Immunoblotting analysis for the detection of representative hepatocyte marker genes using the total cell lysates in vitro cultured HLCM-HH, hepatoma cell lines (Huh7 and HepG2 cells), NTCP-overexpressing hepatoma cells, normal human liver tissue from three independent donors (D1-D3). **E-G.** Cellular susceptibility to HDV infection was assessed using indicated cell types via IFA (E), immunoblotting (F), and RT-qPCR analysis (G). The infection was carried at low (500 GEq/cell) and high (5000 GEq/cell), which correspond approximately to MOI 4 and 40, respectively, for 7 days. Scale bar: 100µm. The % shown in the image indicates the median HDAg positive foci/total number of cells. Results are shown as mean ± SD of triplicate samples. $^{*}P < 0.05$, $^{**}P < 0.01$, $^{***}P < 0.001$ were determined by one-way ANOVA with post-hoc Tukey test. **H.** Representative image of EMCV cytopathic effect assay using Huh7 cells. Cells were first incubated in the presence of increasing concentration of IFN-β (0, 1, 5, 10, 20, 100, 1000 IU/ml) or IFN-λ (0, 1, 5, 10, 20, 100, 1000 ng/ml) for 24 hours prior to the inoculation with increasing titer of EMCV at 0, $1x10^2$, $5x10^2$, $1x10^3$, $5x10^3$, $1x10^4$ PFU/ml for 48 hours followed by crystal violet staining. **I.** RT-qPCR analysis of fold changes in expression of the indicated ISGs in PHH (three independent donors D1-D3) infected with HDV (5000 GEq/cell) for 7 days. SeV (100 HAU/ml) 24 hours serves as the positive control. Results are shown as mean ± SD of

triplicate samples. Displayed data represent one of the biological triplicate experiments (A-H).

Supplemental Figure 2. A-B. PCA analysis of mRNA sequencing data of HLCM-HH infected with HBV or HBV-HDV co-infection (A) and the Venn diagram illustrates the DEG in HBV mono-infected (left) and HBV-HDV coinfected cells (B). The numbers in black, blue, and red represents the DEG, IRG, and ISGs in each category, respectively. **C-D.** mRNA sequencing analysis of HLCM-HH infected with HDV (5000 GEq/cell) for 7 days. The hierarchical clustering demonstrates the DEGs (cutoffs used were a P value of 0.01 and a fold change of 2) (C), and the Venn diagram illustrates the ISGs included in the DEG of HDV mono-infected (left) and HBV-HDV co-infected HLCM-HH (right). **E.** HLCM-HH were incubated in the presence of increasing concentration of TOF for 10 days followed by light microscopic observation (top) as well as Hoechst 33342 Fluorescent staining (bottom). Scale bar: 100μm. **F-G.** HLCM-HH treated with indicated types IFN (0, 0.1, 1, 10, 100 or 1000 IU/mL) for 2 or 8 hours in the presence of TOF (10 μM) were subjected to immunoblotting or RT-qPCR analysis of indicated molecules, respectively. Results are shown as mean ± SD of triplicate samples. **H.** Culture supernatant and cell lysate of HCLM-HH infected with indicated genotypes of HBV (MOI 50) either as a mono-infection or coinfection with HDV (5000 GEq/cell) for 10 days were subjected to quantification of HBV DNA and HDV RNA as well as relative expression changes of indicated ISGs via RT-qPCR. Results are shown as mean ± SD of triplicate samples. *$P < 0.05$, **$P < 0.01$, ***$P < 0.001$ were determined by one-way ANOVA with post-hoc Tukey test. Displayed data represent one of the biological triplicate experiments (E-H).

Supplemental Figure 3. A-B. HLCM-HH either mono-infected with HBV or superinfected with HBV-HDV in the presence of TOF (10μM) for 10 days and the culture supernatant were subjected to RT-qPCR analysis for the quantification of HBV DNA (A) and HDV RNA (B). **C.** HLCM-HH were first either mock or mono-infected with HDV for 5 days followed by superinfection with either mock or HBV for an additional 10 days as shown the infection schedule above the bar graphs, and the culture supernatant were subjected to RT-qPCR analysis for the quantification of HBV DNA (left) and HDV RNA (right), respectively. Results are shown as mean ± SD of triplicate samples. *P < 0.05, **P < 0.01, ***P < 0.001 were determined by one-way ANOVA with post-hoc Tukey test. Displayed data represent one of the biological triplicate experiments (A-C).

Supplemental Figure 4. A. HCLM-HH mono-infected with HBV, superinfected with HBV-HDV for 10 days, or mono-infected with HDV for 7 days followed by PegIFNα-2a (10 ng/ml) treatment for indicated hours, and cell lysates were subjected to RT-qPCR analysis of indicated ISGs. Results are shown as mean ± SD of triplicate samples. **B-C.** Immunoblotting and IFA analysis of HLCM-HH transduced with lentiviral particle encoding control (empty vector: EV), S-HDAg, or L-HDAg for 7 days. Mock or HDV infected HLCM-HH for 7 days were included as negative and positive control, respectively. **D-E.** HLCM-HH stably expressing control EV, S-HDAg, or L-HDAg via lentiviral transduction for 7 days were treated with IFN-β (100 IU/ml) or SeV (100 HAU/ml) for 16 hours and then total cellular RNA were subjected to RT-qPCR analysis for the relative quantification of indicated genes. Results are shown as mean ± SD of triplicate samples. *P < 0.05, **P

< 0.01, ***P < 0.001 were determined by one-way ANOVA with post-hoc Tukey test. Displayed data represent one of the biological triplicate experiments (A-E).

Supplemental Figure 5. Outline of in vivo study design. HLCM (16-26 weeks old: n=40) were allocated to mock (PBS injection at week 0: n=12), HBV mono-infection (HBV GTA, 10^6 copies/animal at week 0: n=12), and HBV-HDV superinfection (HBV GTA, 10^6 copies/animal at week 0 and HDV 10^7 gEq/animal at week 7: n=16), which were then killed for sampling 24 hours or 7 days after PBS or PegIFNα-2a (30ng/g) treatment.

Supplemental Material & Method Table 1

qPCR primer sequences for the gene expression analysis

Gene Symbol	Forward	Reverse
**CXCL10**	GCCATTCTGATTTGCTGCCT	TTGATGGCCTTCGATTCTGGA
**IFI44L**	TGCACTGAGGCAGATGCTGCG	TCATTGCGGCACACCAGTACAG
**IFI6**	TGATGAGCTGGTCTGCGATCCT	GTAGCCCATCAGGGCACCAATA
**IFITM1**	ACTCCGTGAAGTCTAGGGACA	TGTCACAGAGCCGAATACCAG
**ISG15**	CAAATGCGACGAACCTCTGA	CCGCTCACTTGCTGCTTCA
**OAS1**	TGTCCAAGGTGGTAAAGGGTG	CCGGCGATTTAACTGATCCTG
**RSAD2**	CCAGTGCAACTACAAATGCGGC	CGGTCTTGAAGAAATGGCTCTCC
**IFN-β**	ACCAACAAGTGTCTCCTCCA	AAGCCTCCCATTCAATTGCC
**IFN-λ1**	GGACGCCTTGGAAGAGTCACT	AGAAGCCTCAGGTCCCAATTC
**IFN-λ2/3**	AGTCGCTTCTGCTGAAGGAC	TCCAGAACCTTCAGCGTCAG
